# Combining models to generate consensus medium-term projections of hospital admissions, occupancy and deaths relating to COVID-19 in England

**DOI:** 10.1098/rsos.231832

**Published:** 2024-05-22

**Authors:** Harrison Manley, Thomas Bayley, Gabriel Danelian, Lucy Burton, Thomas Finnie, Andre Charlett, Nicholas A. Watkins, Paul Birrell, Daniela De Angelis, Matt Keeling, Sebastian Funk, Graham Medley, Lorenzo Pellis, Marc Baguelin, Graeme J. Ackland, Johanna Hutchinson, Steven Riley, Jasmina Panovska-Griffiths

**Affiliations:** ^1^ UK Health Security Agency, London, UK; ^2^ MRC Biostatistics Unit, University of Cambridge, , UK; ^3^ Department of Mathematics, University of Warwick, Coventry, UK; ^4^ London School of Hygiene and Tropical Medicine, London, UK; ^5^ University of Manchester, Manchester, UK; ^6^ Imperial College London, London, UK; ^7^ University of Edinburgh, Edinburgh, UK; ^8^ Queen’s College, University of Oxford, Oxford, UK; ^9^ The Big Data Institute and the Pandemic Sciences Institute, University of Oxford, Oxford, UK

**Keywords:** SARS-CoV-2, modelling, COVID-19 medium-term projections (MTPs), statistical modelling, ensemble modelling

## Abstract

Mathematical modelling has played an important role in offering informed advice during the COVID-19 pandemic. In England, a cross government and academia collaboration generated medium-term projections (MTPs) of possible epidemic trajectories over the future 4–6 weeks from a collection of epidemiological models. In this article, we outline this collaborative modelling approach and evaluate the accuracy of the combined and individual model projections against the data over the period November 2021–December 2022 when various Omicron subvariants were spreading across England. Using a number of statistical methods, we quantify the predictive performance of the model projections for both the combined and individual MTPs, by evaluating the point and probabilistic accuracy. Our results illustrate that the combined MTPs, produced from an ensemble of heterogeneous epidemiological models, were a closer fit to the data than the individual models during the periods of epidemic growth or decline, with the 90% confidence intervals widest around the epidemic peaks. We also show that the combined MTPs increase the robustness and reduce the biases associated with a single model projection. Learning from our experience of ensemble modelling during the COVID-19 epidemic, our findings highlight the importance of developing cross-institutional multi-model infectious disease hubs for future outbreak control.

## 1. Introduction

Mathematical modelling has played an important role in offering scientific advice to policymakers at important junctions over the COVID-19 pandemic. Modelling allows information from data and epidemiological theory to be used in a transparent and rigorous technical framework, within which current epidemic trajectories can be assessed and projections of future behaviours can be made. Epidemiological estimates of how a virus may spread in the future along with uncertainties and limitations surrounding these estimates are useful tools for future policy planning.

Over the COVID-19 epidemic, a number of models have been developed and used at pace. Their main power is in populating the technical framework with data and generating (probabilistic) projections of possible futures. While fitting to historic data and processes^1^ gives tight constraints on the models’ behaviour in the past, projecting trends into the future can be uncertain and dependant on the assumptions of the models about future events such as schools opening/closing, level of social mixing or the intrinsic properties of the circulating virus which enable it to escape imposed intervention and vaccination mitigation strategies. Specifically, when modelling the future, modellers are faced with complex and uncertain scenarios owing to the high dimensionality of the system being modelled in terms of free parameters and compartments, as well as the possibility of multiple entangled causal chains. The decision-making process relies on them making assumptions on what may happen in the future and quantifying the likelihood of those potential outcomes [1,2].

Ensemble modelling is a quantitative method that combines information from multiple individual models to generate a combined or consensus outcome. It is a common practice in climate science [3–5], economics [6] and weather forecasting [7,8]. Similarly to climate modelling, uncertainties in epidemic model outputs arise from uncertainties in initial conditions, recorded observations, model assumptions and model structure. Since different models have different underlying assumptions and hence project different possible futures, aggregating and combining results from a number of models may mitigate some of the uncertainty of the possible futures. There are also uncertainties in the model parameters and structural uncertainties resulting from the fact that some processes in the modelled system are not fully understood, or are impossible to model completely owing to computational constraints. Using a model ensemble can help to characterize the overall uncertainty in a system [9]. Furthermore, averaging the outputs of multiple model ensembles has been shown numerous times to compare more favourably with observations and yield better projections than a single model [10].

Combining epidemiological models to generate aggregated model outcomes pre-COVID-19 was applied to modelling HIV [11], influenza [12], Ebola [13,14] and Dengue [15] transmission. However, this has developed rapidly over 2020–2022 with a number of countries setting up modelling hubs to ensemble model the COVID-19 epidemic [16–18].

In the United Kingdom, such a modelling hub was formed as a formal collaboration between the Department of Health and Social Care (DHSC) advisory committee on pandemic modelling (Scientific Pandemic Influenza Group on Modelling—Operational (SPI-M-O)) and the UKHSA Epidemiological Ensemble group (UKHSA Epi-Ensemble). Since early 2021, this modelling hub has provided the UK government with weekly medium-term projections (MTPs) as a combined epidemic trajectory estimate from a number of epidemiological models (having taken over from SPI-M-O, which produced the MTPs before this in 2020). These comprise epidemic trajectories of hospital admissions, hospital bed occupancy and deaths over the future 4–6 weeks of the epidemic and are generated from a set of epidemiological models maintained and run by members of SPI-M-O or the UKHSA Epi-Ensemble.

Production of the MTPs began in late August 2020 and was initially generated by SPI-M-O on a weekly basis [19], to replace the short-term forecasts that had been generated up to that point several times a week [20]. With the development of the SPI-M-O and UKHSA Epi-Ensemble modelling hub, the responsibility to produce MTPs was transferred to the UKHSA Epi-Ensemble in early 2021. As part of the collaboration, modelling teams within SPI-M-O and UKHSA Epi-Ensemble were asked to produce projections under an explicit assumption of no changes other than population immunity [21]. This was done to allow policymakers to assess the likely outcomes of the epidemic based on current policy interventions. While the modelling teams were provided with expected vaccination rates and asked to incorporate known factors such as school term times, in general, the MTPs were not planned to be forecasts. Therefore, evaluating them as such deviates from their original purpose. However, the analyses undertaken here give valuable retrospective insight into the capability of models used during a pandemic, which is useful for future pandemic preparedness planning. Furthermore, the time frame considered here almost entirely coincides with there being no legal COVID restrictions, so the differences between predictions and projections for the purposes of this analysis are minimal.

As we discussed in our previous paper [22], the value in getting a combined forecast from across models and datasets is not only just in the weighted averaging of those estimates but also in the formation of a community that is constantly discussing the outcomes, the modelling assumptions and the input data, identifying the drivers behind the differences across models’ outcomes when formulating the aggregated possible future projections.

The United Kingdom was not the only country to produce COVID-19 forecasts from a model ensemble. For example, modellers in the United States, in conjunction with the Center for Disease Control (CDC), published ensemble forecasts using a wide variety of mathematical models [2,23] forecasting new cases, hospitalizations and deaths at a national and state level as part of both the US COVID-19 Forecast Hub and the Scenario Modelling Hub (SMH). The predictive performance of the former, which was formed in April 2020, was evaluated for the period June 2020 to October 2021 for deaths only, at both national and state levels using similar methods to this article [24]. The output performance of the SMH, which was formed in December 2020 to produce longer-term scenario forecasts, was evaluated for the period between February 2021 and November 2022, and takes into account both scenario plausibility and model calibration [25]. Similarly, the European Center for Disease Control (ECDC) developed a public European COVID-19 Forecast Hub, the output performance of which was evaluated for cases and deaths across Europe between March 2021 and March 2022 [26].

In light of the above, this article outlines how MTPs were generated in England using a previously established combination method [27] throughout the COVID-19 pandemic, as a collaboration across government and academia. We detail the approach of generating a single, combined consensus model projection from an ensemble of multiple epidemiological models applied to the English epidemic, and how they were combined to produce aggregated MTPs over time. We follow this with a detailed evaluation of the performance of the generated combined estimates over the period November 2021–December 2022, noting when the MTPs were better or worse at estimating the current epidemic status and projecting forward trajectories and exploring why.

## 2. Methodology

### 2.1. Models used to produce medium-term projections

A number of mathematical models have been developed, adapted and used throughout 2020–2022 to model the COVID-19 epidemic and produce MTPs in England. The models described in this article broadly fall into three groups: population-based (PBMs), agent-based (ABMs) and data-driven models (DDMs). Appendix A contains the models in the ensemble, with a description of their main characteristics.

### 2.2. Data sources

Data sources are important in modelling—both in parametrizing the models and in calibrating/validating them. In this ensemble, the models were informed by a range of different data sources and were fitted to specific data for the projections of each metric. In the data that were used, and therefore for the purposes of this article, the metrics are defined in table 1. These data were provided to the modellers via a secure transfer from internal sources. A summary of the data to which each model is fitted is described in appendix A.

**Table 1.** The definitions of the metrics used from the data source.

metric	data definition
hospital admissions	daily number of inpatients with a positive diagnosis of COVID-19
hospital bed occupancy	daily number of hospital beds occupied with confirmed COVID-19 patients
deaths	daily number of people with laboratory-confirmed positive COVID-19 tests, who died within (equal to or less than) 28 days of the first positive specimen date

### 2.3. Collaborating across government and academia to produce a consensus medium-term projection

The collaboration between academia and government to produce MTPs largely mirrored the procedures outlined in §2.3 of our earlier publication for the production of the R number. As with the combined R , a combined projection would be produced for a given metric provided there were three or more constituent models, and any individual model would be accepted in the combination unless it demonstrated behaviour inconsistent with epidemiological principles or if there was a clear error with the model fit. The MTP production also required a decision on the projection length, the standard for which was either 4 or 6 weeks, as predictive performance sharply decreased beyond the 6-week upper limit. However, at times of significant uncertainty, for example, in the early stages of the Omicron variant emerging, a collective decision was made to publish projections with shorter forecast horizons. Once a consensus was reached, both the individual and combined model outputs were provided to decision-makers for transparency.

### 2.4. Combining model projections to generate a consensus medium-term projection

Each of the calibrated epidemiological models described in appendix A were able to produce MTPs for one or more of: hospital admissions, hospital occupancy and deaths over the future 4–6 weeks. The results from the modelling groups were submitted as quantiles of the posterior predictive distribution of the model’s outcomes, with quantiles ranging from the 5th to 95th in increments of 5. Posterior predictive distributions were then estimated for each model as skewed-Normal distributions fitted to the submitted set of quantiles [27,28].

To illustrate how these distributions were then combined, we can consider a list of epidemiological models $M=(M_{1},\;M_{2},\;...\;,\;M_{N})$ , which are fitted to observed data y and generate projected data f , where the nth model has a posterior predictive distribution $p_{n}(f|y)$ , the model ensemble will then have a posterior predictive density of the form


$$p(f|y)=\sum_{n=1}^{N}w_{n}p_{n}(f|y).$$

In the above, $w_{k}\geq 0$ weights the kth model’s contribution to the ensemble, and $\sum_{n=1}^{N}w_{n}=1$ . For the combined projections, we used an equal weights combination method, setting $w_{n}=w=\frac{1}{N}$ . The visualization of the projections was implemented using CrystalCast [29], a piece of software developed by the Defence Science and Technology Laboratory (DSTL).

### 2.5. Assessing model performance

The performance of each model can be assessed quantitatively by evaluating the point and probabilistic accuracy of the individual model projections. We examined the predictive performance for England for a range of forecast horizons from $t\in [t_{\text{0}},t_{\text{0}}+7]$ to $t\in [t_{\text{0}},t_{\text{0}}+28]$ to highlight how the accuracy changes for different projection lengths, where $t_{\text{0}}$ is the date of the first day of the forecast. For all of the models, data were available until $t_{0}-t_{-5}$ days, and each model was fitted to data from the same source. However, on certain weeks, there were issues with data availability, meaning data were only available up to an earlier date. At this point, modellers were faced with a decision on what time period from the data to include, and this was left to modellers’ discretion. Some models used the data to the last available date, whereas some truncated a few days earlier. This led to temporal heterogeneity at these specific time points, generally in the instance of a new variant or a sudden change to the epidemic dynamics. The methods we used to evaluate and compare the predictive accuracy of the models are detailed below.

#### 2.5.1. Mean absolute error

The absolute error (AE) was evaluated for each point of the projections. For a given model, this is the absolute difference between the projected median and the recorded data time series. The mean absolute error (MAE) then gives the mean of this value for a given projection, taken over the entire forecast horizon. The MAE is useful as it provides an intuitive, quantitative estimate of the forecast performance in the natural units of the data. To calculate this, we used the standard formula:


$$\text{MAE}=\frac{1}{N}\sum_{i=1}^{N}|y_{i}-x_{i}|,$$

where N is the length of the forecast horizon in days, $y_{i}$ is the central estimate of the projection and $x_{i}$ is the recorded data on day i . We calculated the MAE for each published MTP for each metric, to examine the trend across the time period and observe how the forecast accuracy changes with successive Omicron variants.

#### 2.5.2. Weighted interval score

We also assessed the probabilistic accuracy using a scoring rule. There were many proper scoring rules at our disposal from the literature [30]. For this analysis, we used a weighted interval score (WIS) [17], aggregated over the forecast horizon:


$${\text{WIS}}_{\alpha_{0:K}}=\frac{1}{N}\,\sum_{i=1}^{N}\left(\frac{w_{0}\,\left|y_{i}-m\right|+\sum_{k=1}^{K}w_{k}\,{\text{IS}}_{\alpha_{k}}\,\left(f_{i},y_{i}\right)}{K+\frac{1}{2}}\right),$$

where K is the number of quantiles being evaluated, $w_{0}$ is the weight given to the median and m is the predictive median. As above, $f_{i}$ and $y_{i}$ are the projected and observed data, respectively, on day i . ${\text{IS}}_{\alpha }(f_{i},y_{i})$ is the interval score on day i , given by


$${\text{IS}}_{\alpha }(f_{i},y_{i})=(u_{i}-l_{i})+\frac{2}{\alpha }(l_{i}-y_{i})1_{\left\{y_{i}<l_{i}\right\}}+\frac{2}{\alpha }(y_{i}-u_{i})1_{\left\{y_{i}>u_{i}\right\}}\text{.}$$

Here, 1 is the indicator function, meaning that $1_{\left\{y_{i}<l_{i}\right\}}=1$ if $y_{i}<l_{i}$ and 0 otherwise. The terms $l_{i}$ and $u_{i}$ denote the α/2 and 1−α/2 quantiles of $f_{i}$ . The first term in the interval score gives the sharpness of the forecast, and the latter two are penalty terms for observations falling below $l_{i}$ and above $u_{i}$ , respectively. For this analysis, we set $w_{0}=1/2$ and $w_{k}=\alpha_{k}/2$ as suggested in the literature [17]. The use of the WIS was chosen as, unlike the MAE, it takes into account the confidence intervals (CIs) as well as the central estimates of the projections, and can give us values for the score at each time step. A projection will receive a good (small) WIS score if central estimates sit close to the observed data and CIs surrounding the central estimate are both narrow and cover all the true data. We calculated the WIS for each time step of the MTPs and took the mean to get an overall score of the projection similar to the MAE.

#### 2.5.3. Weighted interval score on the log scale

We also calculated the WIS of the log-transformed data, by taking the natural logarithm of both the recorded data and forecasts before scoring. This can be seen as an approximation of a probabilistic counterpart to the symmetric absolute percentage error (SAPE), by considering the absolute error of the log-transformed data, $\epsilon^{*}$ :


$$\left|\epsilon^{*}\right|=\left|\log\left(f\right)-\log\left(y\right)\right|=\left|\log\left(f/y\right)\right|,$$

using the Taylor expansion for log⁡(f/y) and, assuming that f≈y , we can approximate the absolute error, $|\epsilon^{*}|$ :


$$|\epsilon^{*}|\approx |f/y-1|\approx |\frac{f-y}{y}|\approx |\frac{f-y}{y/2+f/2}|.$$

The alignment with SAPE has been shown to hold reasonably well even if the predicted and observed values differ by a factor of 2 or 3 [31]. In the rest of this article, we will refer to the WIS of the log-transformed data as log WIS, for brevity.

#### 2.5.4. Relative weighted interval score

Following [24], we calculated the relative weighted interval score (rWIS) for each model as the geometric mean of the pairwise relative WIS. For a pair of models $m_{i}m_{j}$ , the pairwise rWIS for each projection round is calculated as:


$$\theta_{m_{i}m_{j}}=\frac{\text{mean WIS of model }m_{i}}{\text{mean WIS of model }m_{j}},$$

then the rWIS for each model for a given projection round is found by taking the geometric mean:


$$\text{rWIS }={\left(\prod_{m_{j}=1}^{M}\theta_{m_{i}m_{j}}\right)}^{1/M}.$$

#### 2.5.5. Empirical coverage

For a forecast horizon, h , and projection interval width, 1−α , the empirical coverage of a model (often referred to also as calibration [32]) is calculated as the proportion of forecast targets (across all forecast dates) for which the projection interval contained the true value; a well-calibrated model has empirical coverage equal to the width of the nominal projection interval (i.e. the 50% projection interval should contain the true value 50% of the time). We calculated the empirical coverage for the 50% and 90% projection intervals over a range of forecast horizons from 1 to 21 days.

#### 2.5.6. Sharpness

Sharpness measures how good a model is at producing narrow (sharp) projection intervals. We measured sharpness as the weighted sum of the width of the 50% and 90% projection intervals, choosing weights of $w_{k}=\alpha_{k}/2$ as we did for the WIS calculation, and again aggregating over the forecast horizon:


$$\text{sharpness}=\frac{1}{N}\,\sum_{i=1}^{N}\sum_{k=1}^{K}\frac{\alpha_{k}}{2}\,\left(u_{\alpha_{k}}^{i}-l_{\alpha_{k}}^{i}\right),\alpha_{k}\in \left[0.5,0.1\right],$$

where K is the number of quantiles being evaluated and $u_{\alpha_{k}}^{i}$ and $l_{\alpha_{k}}^{i}$ are the upper and lower bounds, respectively, for a given quantile $\alpha_{k}$ on day i . We calculated the sharpness for all the models, projected out to a 28-day forecast horizon, against the publishing date. It is worth noting that sharpness is a property of the forecast only. Therefore, we evaluated the predictive performance based on ‘the paradigm of maximizing the sharpness of the predictive distributions *subject to calibration*’ [33] and took the calibration to be directly evaluated by empirical coverage.

#### 2.5.7. Bias

Bias measures a model’s tendency to over- or underpredict. To calculate this, we simply subtracted the sum of recorded observations from the sum of the central model estimate for a given metric and did this for each publishing date.

### 2.6. Forecast comparison

In order to evaluate the utility of the ensemble approach, we compared the predictive performance of the combined MTPs with the performance of the constituent models. We measured the performance by calculating the MAE, WIS, sharpness and empirical coverage of the combined model and its constituents over a range of publication dates and forecast horizons for the three metrics. We did this for MTPs published between November 2021 and December 2022. For the empirical coverage, we aggregated the models over the entire time period and evaluated over a range of forecast horizons. For WIS, sharpness and MAE we evaluated and compared all of the models at 7-, 14-, and where applicable 21- and 28-day forecast horizons for each published MTP over the range of publishing dates.

## 3. Results

### 3.1. COVID-19 hospital admissions, bed occupancy and deaths data between November 2021 and December 2022

**Figure 1.**
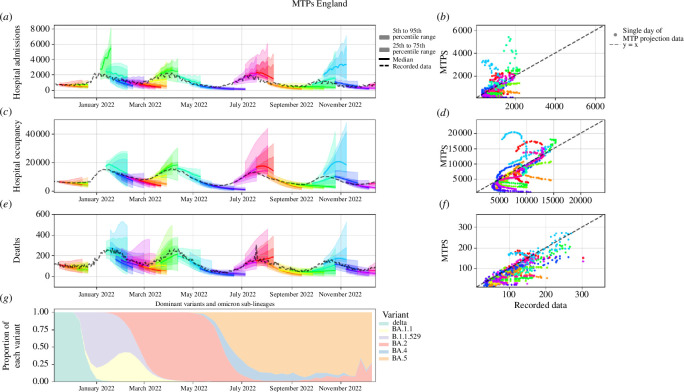
Combined MTPs for hospital admissions, hospital bed occupancy and deaths plotted against the recorded data in (*A*,C,E), respectively, for the period between November 2021 and December 2022. Hospital admissions were the only metric for which a combination was published on 6 January 2022, meaning there is one extra combined MTP shown in (A). (B,D,F) Scatter plots of the forecast hospital admissions, bed occupancy and deaths plotted against the recorded data, where each point represents a single day of data. Each colour in (A*–F*) represents a single published combined forecast, each with a forecast horizon of 28 days. Plot (G) uses data from [34] and shows the relative proportions of cases belonging to each variant and subvariant from the genomes that were sequenced.

Each of the models was able to produce time series for at least one of hospital admissions, bed occupancy and deaths related to COVID-19. The time series for the model ensemble are shown in figure 1*a*,*c*,*e*. Hospital admissions, bed occupancy and deaths were all relatively low at the tail end of 2021, reaching a 7-day average trough of 643 new hospital admissions, 5900 beds occupied and 95 deaths on 25 November, 4 and 10 December, respectively. At this time, the Delta (B.1.617.2) variant was still responsible for the vast majority of cases [34]. In late November 2021, the first Omicron cases were detected in the United Kingdom, onsetting the first Omicron ‘wave’, which saw infections increase before peaking with a 7-day average of 2040 new hospital admissions, 16 696 beds occupied and 253 deaths on 1, 12 and 19 January 2022, respectively. This wave consisted of a mix of BA1.1 and B1.1.529, visible in figure 1*g*, which shows the relative proportions of the variants over time. All legal COVID restrictions were officially lifted in England on 24 February 2022. In late March, booster vaccinations were offered by NHS England to people aged over 75 and anyone over the age of 12 who was considered medically vulnerable. Around the same time the BA.2 Omicron sub-lineage led to the second Omicron wave in spring and early summer, which peaked with 2116 new hospital admissions, 16 600 beds occupied and 250 deaths on 28 March, 7 and 10 April, respectively. On 1 July, the UK government dashboard moved from daily to weekly reporting [35]. The BA.4 and BA.5 sub-lineages co-existed throughout autumn 2022, as shown in figure 1, which helped drive the third Omicron wave that caused 7-day averages of 1864 new hospital admissions, 13 849 beds occupied and 189 deaths on 10, 16 and 18 July, respectively. The wave at the end of the year consisted of a mixture of previously established Omicron sub-lineages, namely BA.2, BA.4 and BA.5. This wave peaked with 7-day average counts of 1212 new hospital admissions, 10 560 hospital beds occupied and 152 deaths on 4, 15 and 19 October, respectively.

### 3.2. Evolution of medium-term projections

Plots showing the combined model projections are given in figure 1. The left-hand column shows how the MTPs evolve over time, with each colour in the plot representing a specific publishing date. The right-hand column shows the central estimate of the combined model plotted against the observed data, with a y=x line for comparison. Each point in the plot represents the projected value for each time step of the projected data. A perfect fit to the data would be along the y=x line in this plot. Therefore, the perpendicular distance of points from this line shows the discrepancy between the published MTPs’ central estimate and the recorded data.

From figure 1, we can see that overall the combined MTPs were in good agreement with the data for most of the Omicron epidemic waves. The MTPs were able to predict the trends of increasing and declining epidemic curves, although the visual inspection suggests that the combined model generally performed worse when forecasting hospital bed occupancy during the Omicron sub-lineage waves in the summer and autumn of 2022. The trend is less clear for hospital admissions and deaths. The 90% CIs are widest when projecting over the epidemic peaks. Figure 1*g* shows a stacked area plot of the proportions of each variant over time from genomic sequencing data [34].^2^

### 3.3. Forecast evaluation

#### 3.3.1. Combination

**Figure 2.**
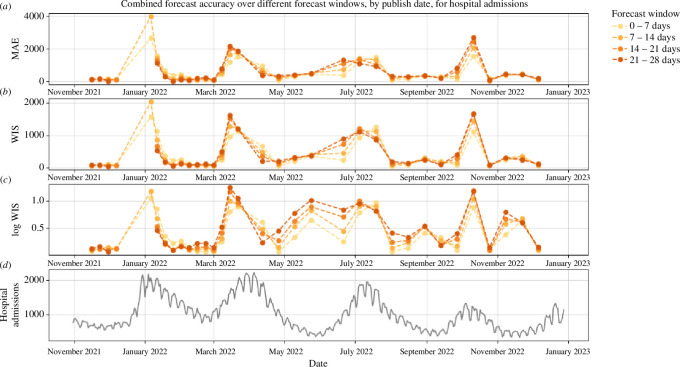
MAE and WIS of the combined MTPs, for hospital admissions, during the period between November 2021 and December 2022. The different colours represent different parts of the forecasting window up to 4 weeks. In some instances, a combined projection was not published beyond 2 weeks (i.e. in January 2022), hence there are some publishing dates which do not have plotted values for longer forecast horizon windows. Plot (A) shows the MAE for the naturally scaled data, and plots (B) and (C) show the WIS for the natural and logarithmic scale, respectively. The observed data are shown in plot (D) for reference.

The combined model had the highest values of MAE, WIS and log WIS for hospital admissions during the first Omicron (B.A.1) wave in January 2022. The mean WIS, log WIS and the MAE reached their maximum on 5 January. Furthermore, the models were only projected out to 2 weeks in this instance,^3^ so we do not have results for forecast horizons beyond 14 days. For hospital bed occupancy and deaths, there were no published combined projections on 5 January. In general, the model ensemble has lower MAE and mean WIS when the number of hospital admissions is either nearing or just past an epidemic peak and has the highest MAE and mean WIS around the time of the epidemic wave peak. The log WIS is lowest when the number of admissions is nearing or just past both epidemic peaks and troughs, and highest around the time the peaks and troughs occur. This suggests that the models perform worse around turning points in general, which is consistent with existing literature [36,37]. Unsurprisingly, the combined model generally has a much lower MAE for the 0–7-day forecast window compared with the more than 7-day forecast windows, with the notable exceptions occurring when hospital admissions had just passed the epidemic wave peak, in early January 2022 and mid-July 2022. The log WIS from May 2022 to July 2022 has a greater dependence on the forecast window than the natural scale score, with the performance being much better for the forecast windows closer to the start date. The MAE, WIS and log WIS for hospital admissions are plotted in figure 2, and similar plots are given for hospital bed occupancy and deaths in the electronic supplementary material.

#### 3.3.2. Individual models

**Figure 3.**
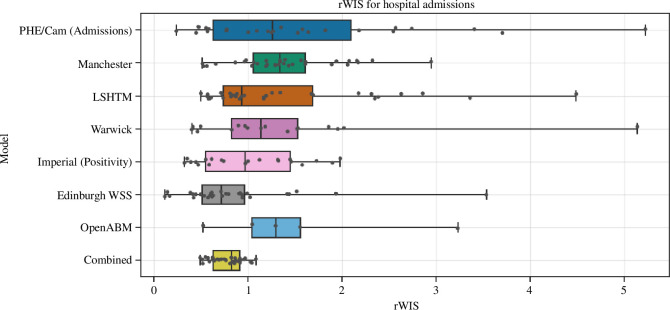
Boxplot for the relative weighted interval score (rWIS) of all models, including the combined model, calculated over the entire time period for hospital admissions. The *x* axis has been scaled to be between 0 and 1.

We found that the predictive ability of each individual model changed over time, and whilst the mean WIS and log WIS of the combination were often not the lowest, no single model consistently outperformed the combination. This is demonstrated by figure 3, which shows the distribution of rWIS values calculated over the whole period. The combined model has a lower range and interquartile range (IQR) than every model in the ensemble and has a lower median than all but one model (Edinburgh WSS). The combined model also has the lowest maximum rWIS value of any model.

**Figure 4.**
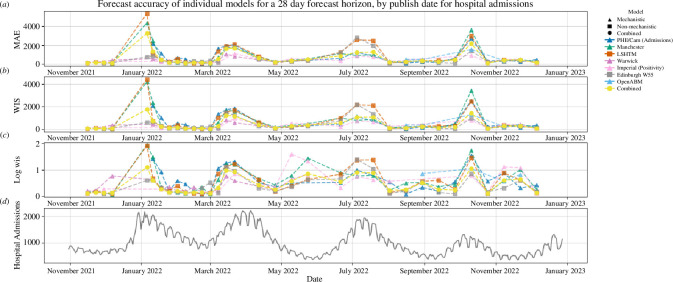
MAE and WIS of the individual models’ submitted MTPs for hospital admissions for the period between November 2021 and December 2022. The MAE and WIS were calculated with a forecast horizon of 28 days. Plot (A) shows the MAE for the naturally scaled data, and plots (B) and (C) show the WIS for the natural and logarithmic scale, respectively.

The individual models are heterogeneous in their predictive ability (figure 4); however, the combination smooths out many of these heterogeneities (figure 3) and is therefore more robust to changes in the status of the epidemic than any of the individual models. The log WIS shows the benefit of a model combination more clearly around the epidemic troughs when compared with the natural scale. In the periods from February 2022 to March 2022 and from May 2022 to July 2022 the combined model has a log WIS of nearly half that of the worst performing models (figure 4).

#### 3.3.3. Empirical coverage, sharpness and bias

**Figure 5.**
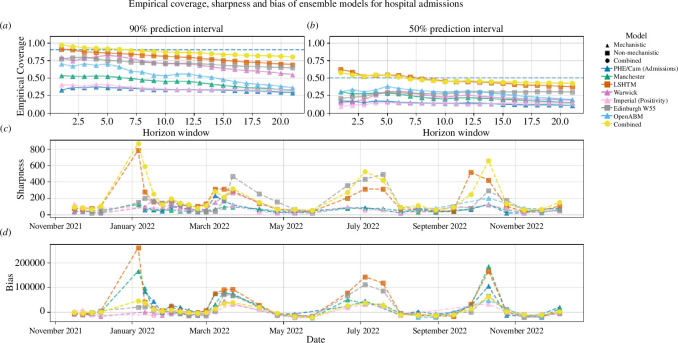
Plots (A) and (B) show the empirical coverage of the individual and combined models for forecasting windows of 1–21 days for the 50% and 90% CIs. Sharpness and bias are shown in plots (C) and (D), averaged over a forecast horizon of 28 days. The plots shown are for hospital admissions only.

The combined model performs the best overall at estimating both the 90% and 50% CIs compared to any single model, albeit with the EpiNow2 model being marginally superior for the 90% CI over very short forecast horizons ( < 4 days) (figure 5*a*,*b*). For hospital admissions, in both cases, the empirical coverage is closest to the target around the 7-day forecast horizon. The combined model is one of the least sharp compared with the individual models across the majority of publishing dates (figure 5*c*). However, the models which are consistently the ‘sharpest’ for hospital admissions (i.e. Manchester, Imperial and PHE/Cam (admissions)) also have the lowest empirical coverage.

All of the models in the ensemble have a tendency to overpredict, demonstrated by the bias being positive far more often than negative (figure 5*d*). This effect is greatest around the epidemic peaks. Some underprojection does occur at the troughs, but the effect is much smaller.

Equivalent plots for hospital bed occupancy and deaths are shown in the electronic supplementary material. For occupancy, the empirical coverage of the combined model is overestimated for the 90% CI at forecast horizons lower than 12 days, but the combination has the best empirical coverage for forecast horizons beyond this. For the 50% CI, the combined model has the best empirical coverage beyond 7-day forecast horizons. For deaths, the empirical coverage is overestimated by the combined model as, for both 50% and 90% CIs, more than 50% and 90% of the recorded data points are covered, respectively. Despite this, the combination still has a better empirical coverage than any single model in the ensemble.

## 4. Discussion

This article showcases the process of ensemble modelling of the Omicron epidemic waves over the period November 2021–December 2022. We illustrated how MTPs can be derived from a model ensemble with three groups of models: PBMs, ABMs and DDMs. Additionally, we evaluated the probabilistic accuracy of combined and individual MTPs over this period.

Our results suggest that the combined MTPs were in general the most accurate future projections (as measured by the mean MAE, WIS, log WIS and rWIS metrics) across different forecast windows and time points, as they were able to buffer and overcome the variable accuracy of individual models. They also suggest that the combined model had a more accurate estimation of uncertainty, shown by the accuracy of the empirical coverage of the combination compared with the individual models. The ensemble MTPs were aligned with the data for most of the study period, with this alignment better over periods of exponential increase or decline in the epidemic than around the epidemic peaks and troughs.

Ensemble modelling, although common practice in climate science [3–5], is a developing area in epidemiological modelling. The approach in epidemiological modelling in the pre-COVID-19 era was often focused on developing a specific disease or intervention question, albeit with notable modelling efforts present in HIV [11] and influenza epidemiology [12] and in the response to the Ebola outbreak [13,14]. During the COVID-19 epidemic, with the vast popularization of modelling, a large number of models were being developed using similar assumptions and were parametrized and/or calibrated to the same or similar data sources. This, therefore, lent itself to ensemble modelling. Our findings support the notion that variability emerging from different model parameters, assumptions and structures can be reduced by using a combined estimate, which characterizes the overall uncertainty in a system better than any single model and that a model ensemble produces more accurate forward projections.

While we only published the combined estimate during the epidemic, the individual model projections were available to policymakers and were discussed with them in weekly meetings. For transparency and confidence in the ensemble results, we advise in future epidemics both the individual and the combined estimates are available.

We note that the work presented here is intended to be a retrospective look at the modelling ensemble that generated the MTPs for COVID-19 in England, and which was a joint effort of a number of independent teams, using different modelling methods and modellers’ specific skills and using different additional datasets, among many factors. The intention of this work was not to thoroughly discuss the desirable properties of a good set of models or indeed, how many of a certain structural or data-fed type constitute a necessary or sufficient set, as this would require a large-scale statistical exercise that is beyond the scope of this article. We plan to explore this in the future and specifically investigate how the quantitative considerations of heterogeneous weightings would change the combined estimate. It will also be interesting to explore the necessary proportions of each model type required for a robust combined estimate and whether it is beneficial for the ensemble model to include several projections from the same modelling group, albeit from structurally different models, or distinct scenarios from the same model.

### 4.1. Strengths of our work

One of the strengths of our approach is that we use a variety of models: those that use one type of data or a number of data streams or those that include mechanisms or not. While the ensemble models can be broadly stratified into the three structure-based groups of PBMs, ABMs and DDMs, they are all technically autonomous and have been continuously developed over the pandemic period. Furthermore, we note that although models have aimed to include similar assumptions, there are subtle differences between them. For example, models such as EpiNow2 are data-driven and do not explicitly model mechanisms such as waning immunity or the depletion of the susceptible pool, but incorporate their effect when fitting to data. Mechanistic models such as Imperial’s sircovid or PHE/Cambridge explicitly model the mechanisms of disease spread and include certain interventions such as vaccination rollouts and school-term times. Having these different models in the ensemble not only enriches the variety of outcomes, but also increases the accuracy of the uncertainty measurements for the combined model projections.

Our results for model sharpness and empirical coverage show that the use of a model ensemble increases the uncertainty bounds for the forward-looking projections, while generally containing more data points within the 50% and 90% CIs than any single model. In epidemiological models, the structure of the model and associated assumptions alongside the model parameters determine the characteristics of the resulting projections. For example, the Manchester model requires the modeller to decide on ‘change points’ where the behaviour of viral transmission is changing, achieved by adding a new value for the β parameter which controls the transmission rate. Therefore, if an epidemic trough is approaching in the near future due in part to a waning of immunity in the population, this model is less likely to capture the turning point as it will continue to project the current trend downwards more than another model which explicitly includes waning immunity as a parameter. Equally, the inherent uncertainty in parameters like waning immunity means that two models are highly unlikely to display the same behaviour even if they both model the mechanism directly. This is what adds uncertainty to the model ensemble and allows a wider window of possible outcomes to be generated than if a single model was used. Exemplifying this is the fact that, as mentioned in the previous section, the sharpest models in the ensemble have the lowest empirical coverage. So despite narrow projections, the CIs of these models do not cover the data points as well as the combination. When aiding policy decisions, it is better to have an understanding of the possible futures, and the likelihood associated with each one, rather than have a very confident projection which does not contain the observed data appropriately within its CIs [38].

Another strength of our model ensemble is that we use models that are calibrated to a variety of data sources, detailed in appendix A. Having models that are informed by a variety of data again widens uncertainties in the combined model projections. Furthermore, no data are free of bias so using multiple data sources reduces the bias associated with any single data source. For example, case data are highly sensitive to ascertainment biases, the scale of which can vary over time. Therefore, models that fit to case counts or positivity must be interpreted in the context of testing behaviours and policies at the time. However, admissions data are not free from bias either. The likelihood of being admitted to hospital varies greatly by age. Hence, without age-stratification in the model, it is likely that community transmission is underestimated among younger age groups. Furthermore, the delay between being infected with COVID-19 and being admitted to hospital was on average far greater than that between infection and receiving a positive test, particularly at the time, when free tests were readily available. This presents difficulties when trying to produce timely estimates of community transmission.

### 4.2. Limitations of this work

Our work has some limitations. For example, the constituent models of the model ensemble, which was used operationally, were not consistent over the full time period. Models in the ensemble were being developed continuously, and therefore were subject to ongoing changes. For those models built and maintained by members of SPI-M-O, it was not possible to track all of these changes. Furthermore, the number of models in the ensemble would change for various reasons. This is shown clearly in figure 4, where the OpenABM model is only included in the projections between September 2022 and November 2022. The constantly evolving ensemble makes it more difficult to assess the performance of the ensemble as a whole over the time period, as changes in performance over time—shown by the variable log WIS—can be owing to the specific mix of models at a given time or to the behaviour of the epidemic, and differentiating between the two is non-trivial.

In addition, the models in the ensemble were combined using equal weights stacking. It was beyond the scope of this work to explore alternatively weighted combination methods, but this is something we are planning on undertaking in the future. Specifically, future work would focus on a subset of the models over a shorter period to enable a more even comparison, as well as using an alternative combination and weighting strategies. In order to be operationally viable an alternative weighting would, however, need to be adaptive, in order to incorporate different models entering or leaving the ensemble.

Also, as noted in [25], the difference between projections and observations is a complex combination of both the calibration of the projecting model and whether the assumptions made about the future match up to reality. We acknowledge that while there was an attempt at aligning models in both the data they used and the modelling assumptions that they made, there were a number of model-specific assumptions that needed to be made by the modellers on an individual basis. For example, assumptions around the transmissibility of emerging sub-variants, the modelled level of vaccination uptake, the modelling process around gaining and waning of immunity and the effects of behavioural aspects such as school-term times or assumed community mixing levels. Consequently, there is the possibility of the results being confounded by the mismatch of these assumptions across models. Since the intention of the work was not to compare individual models, but to discuss how they can be combined and what was done during the pandemic, we have not delved into more details of individual model assumptions and mechanisms. We intend to have a more detailed look at the assumptions made by each individual model and comparisons between them in future work.

Finally, none of the models in the ensemble picked up on the oscillatory nature of the epidemic (see figure 1). This is to be expected as the models were designed for the medium term of 4–6 weeks, and the oscillation is a longer-term trend with a period of roughly 10 weeks. Future work could therefore look to combine medium-term forecasts similar to those discussed in this article, with longer-term pattern matching or ARIMA-type models, in order to try and more accurately capture the oscillations of the epidemic in its later stages.

## 5. Conclusions

In summary, our results illustrate that the combined MTPs, produced from an ensemble of heterogeneous epidemiological models across different Omicron epidemic waves, were a closer fit to the data than the individual models throughout late 2021 and during 2022. The consistently low rWIS values over the entire period suggest that the combined model is the most reliable when it comes to predictive performance when compared with the individual models. The alignment with the data was best during the periods of epidemic growth or decline, with the uncertainty being largest, and the log WIS being the highest around the epidemic peaks and troughs. Combined MTPs also improve the robustness and reduce the bias associated with an individual model projection. Hence, we advocate the development of formal national and international ensemble modelling hubs for infectious disease modelling as a key step in preparing for the next outbreak or pandemic.

## Data Availability

The data and the code accompanying this study are sensitive. Some details of the statistical model, data and analysis code, with parts redacted to conceal sensitive information, are in part available within the electronic supplementary material. Further details are available from the corresponding authors at reasonable request. Electronic supplementary material is available online at [39].

## Footnotes

^1^
We note that fitting to historic data is not always necessary, and immunity estimates external to the model can also be used as initial conditions.

^2^
The genomics data ‘only includes a subset of UK SARS-CoV-2 sequencing surveillance data and should not be used to estimate frequency of SARS-CoV-2 variants circulating’ [35]; however, it still provides a good estimate of the relative proportion of different variants, and the rough time that each becomes dominant.

^3^
For a discussion about projection horizons, see §2.3.

## Appendix A. Model and data descriptions

**Table 2.** Outline of the epidemiological models used to generate MTPs for the English COVID-19 epidemic. We list the names of the models, as well as their main modelling characteristics and the data to which they are calibrated against. ­

model name	description	model type	data fit to
Manchester (DetSEIRwithNB) [40]	a deterministic compartmental ODE model in which the transmission rate, β , varies step-wise at certain user-defined change points. These change points correspond to policy changes, behavioural changes (e.g. schools returning and lockdowns), or visually assessed changes in the data trends	PBM	hospital admissions, hospital bed occupancy, ICU occupancy and deaths in hospital
Imperial Stochastic Compartmental (sircovid) [41]	the sircovid model implements a series of mechanistic stochastic compartmental models, described by stochastic difference equations	PBM	deaths, hospital admissions, hospital prevalence, tested cases in hospital beds, ICU prevalence and serology data
PHE/Cambridge [42,43]	the PHE/Cambridge model is a deterministic age-structured compartmental model. Different versions of the model have been run throughout the pandemic that fit to slightly different data streams. For this analysis we consider two versions of the model, one that fits to admissions and ONS deaths data. The two models have the same model structure	PBM	two versions are run fitting to cases and hospital admissions separately. Also uses serology data and Google mobility data
Warwick [44]	the Warwick model is a deterministic, age-stratified compartmental ODE model	PBM	hospital and ICU admissions, and COVID-19 positivity rate data
OpenABM [45]	OpenABM is an age-stratified agent-based model with realistic social networks, that allows the user to explicitly model non-pharmaceutical interventions such as lockdowns, testing, quarantine and digital and manual contact tracing	ABM	hospital admissions
LSHTM EpiNow2 [46,47]	EpiNow2 is a data-driven model which jointly estimates the time-varying reproduction number (with a Gaussian process prior) and infections as latent variables. Hospital admissions are projected forward in time, whereas occupancy and deaths are calculated by establishing a relationship between hospital admissions and the respective metric using the most recent month of data available when the model is run	DDM	hospital admissions
Edinburgh WSS [48]	the weight-shift-scale (WSS) model generates epidemic metrics by multiplying by the normalized probability of moving to a given compartment (for that age group), the vaccination rate and effectiveness, and the variant severity	DDM	cases
